# Analysis of Teg41 and PSMα promoter activity using a divergent fluorescent reporter plasmid

**DOI:** 10.1128/msphere.00432-25

**Published:** 2025-10-31

**Authors:** Paul Briaud, Julia R. Tennant, Ryan W. Steere, Richard E. Wiemels, Marvin Whiteley, Ronan K. Carroll

**Affiliations:** 1 Department of Biological Sciences, Ohio University 110002 , Athens, Ohio, USA; 2 School of Biological Sciences, Georgia Institute of Technology 123387, Atlanta, Georgia, USA; 3 Center for Microbial Dynamics and Infection, Georgia Institute of Technology 1372, Atlanta, Georgia, USA; 4 Emory-Children’s Cystic Fibrosis Center, Atlanta, Georgia, USA; University of Wyoming, Laramie, Wyoming, USA

**Keywords:** sRNA, Teg41, PSM, divergent, promoters

## Abstract

*Staphylococcus aureus* is a common commensal of human skin and anterior nares but also a notable pathogen, causing a range of diseases from minor skin infections to serious, often fatal conditions. The global health community considers *S. aureus* a serious threat due to the rise of multidrug-resistant strains, including community-acquired methicillin-resistant *S. aureus*. Effective host tissue invasion by *S. aureus* requires precise spatiotemporal regulation of virulence factor expression, which can be controlled by small RNAs (sRNAs). Our lab has identified over 300 potential sRNAs in *S. aureus* USA300, highlighting their regulatory significance. Recently, our group characterized an sRNA named Teg41, which positively influences transcription from the PSMα promoter, enhancing toxin production and virulence. Teg41, located 203 bp downstream of the *psmα* transcription start site, exhibits relatively stable growth phase expression, a first for sRNAs in *S. aureus*. We demonstrate that the Teg41 and *psmα* promoters respond to different stimuli, and the Teg41 promoter has distinct regulatory regions. Single-cell analysis reveals non-homogeneous Teg41 expression within the bacterial population, with a subset of cells showing high expression. This study introduces new tools for studying divergent promoters and elucidates the Teg41-*psmα* expression relationship.

**IMPORTANCE:**

*Staphylococcus aureus* is a leading cause of human infections and a major public health concern due to rising antibiotic resistance. Understanding how this pathogen regulates its virulence is critical to developing new therapeutic strategies. sRNAs are key regulators of bacterial gene expression, yet most remain uncharacterized. Here, we investigate Teg41, an sRNA that activates expression of the potent PSMα toxins. Using a novel dual-fluorescence reporter system and single-cell analysis, we uncover that Teg41 and its target promoter are regulated independently and heterogeneously across the bacterial population. These findings reveal new insights into sRNA-mediated regulation of virulence and provide innovative tools to dissect complex gene regulatory networks in *S. aureus*.

## INTRODUCTION

*Staphylococcus aureus* is both a commensal organism found on human skin and in the anterior nares, and a significant pathogen. It causes a wide range of diseases, from superficial skin infections to severe, potentially fatal conditions like sepsis, toxic shock syndrome, and endocarditis. Health care institutions worldwide classify *S. aureus* as a serious threat due to the alarming spread of multidrug-resistant strains within communities (1, 2). Community-acquired methicillin-resistant *S. aureus* (MRSA) has seen a notable increase in cases, with societal costs estimated between $1.4 and $13.8 billion per year (3).

For *S. aureus* to effectively invade host tissues, it must regulate the expression of its virulence factors in a spatial and temporal manner. This bacterium relies on numerous proteinaceous elements intricately woven into environmental sensing networks that regulate gene expression by binding to promoter regions. Additionally, bacterial gene expression can be controlled through RNA-based mechanisms. Small RNAs (sRNAs) are a category of RNAs that bind to mRNAs to modify their expression. Our lab has annotated over 300 potential sRNAs in *S. aureus* USA300, which exceeds the number of known proteinaceous transcription factors (4). This highlights the significant role of sRNAs in gene regulation. Despite this, only a few sRNAs have been characterized in detail, though they demonstrate a primordial role in regulating virulence (5–8).

Recently, our group described an sRNA named Teg41, which controls the expression of PSMα toxins and virulence in *S. aureus* (9, 10). We demonstrated that Teg41 can significantly activate the PSMα promoter, leading to increased production of PSMα toxins. The Teg41 locus is located 203 bp downstream from the transcription start site (TSS) of the *psmα* locus. In this study, we examine the relationship between Teg41 and *psmα* expression within the cell to understand the circumstances under which Teg41*-psmα* expression is regulated. Using a novel dual-fluorescence reporter assay, we show that Teg41 displays a relatively stable expression under a variety of conditions. Moreover, we demonstrate that the Teg41 and *psmα* promoters respond to distinct stimuli and do not share the same regulatory regions. Using single-cell analysis, we also show that Teg41 expression is not homogeneous within the population, with a subset of cells displaying high Teg41 expression. In conclusion, this study introduces novel approaches and tools to study divergent promoters and elucidates the relationship between Teg41 and *psmα* expression.

## RESULTS

### The Teg41 promoter exhibits characteristics of constitutive activity

**Fig 1:**
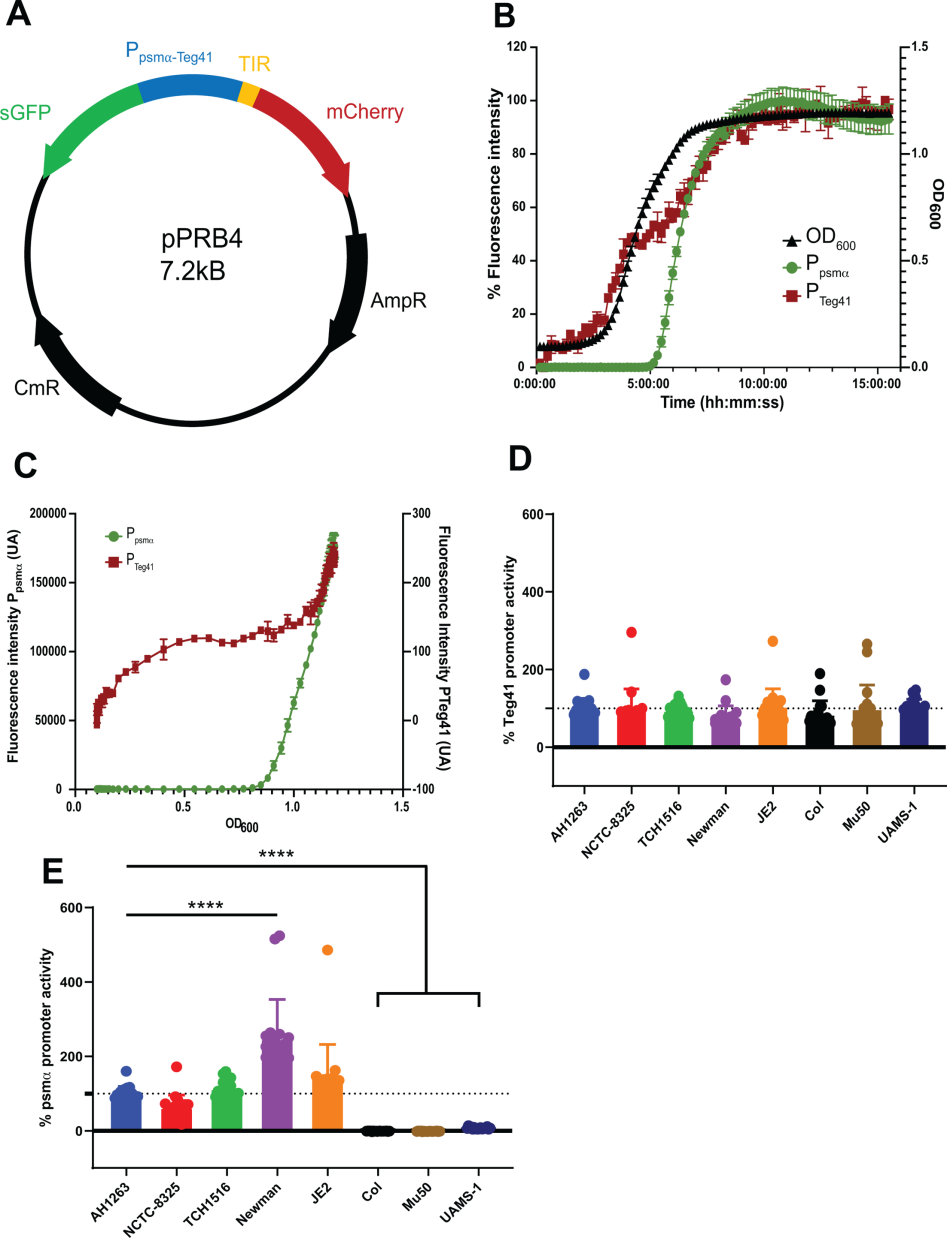
Dual reporter plasmid assay reveals two different activity profiles for the Teg41 and *psmα* promoters. (**A**) Schematic of the dual reporter plasmid (pPRB4) constructed. The *psmα* promoter drives expression of sGFP (green), while the Teg41 promoter controls expression of mCherry (red). A translation initiation region (TIR, yellow) was added before the start codon of mCherry to initiate translation. (**B**) *S. aureus* AH1263 carrying pPRB4 was grown in TSB with chloramphenicol (10 µg/mL) at 37°C, and fluorescence intensities for both sGFP (green) and mCherry (red), as well as OD_600_ (black), were monitored over 16 h. The maximum fluorescence intensity for each fluorophore was set at 100%. Data are represented as the average of three replicates ± standard deviation (SD). (**C**) Relationship between fluorescence intensity and bacterial growth for both the Teg41 (right axis) and *psmα* promoters (left axis). Raw fluorescence data were plotted against bacterial OD_600_. (**D and E**) Plasmid pPRB4 was phage-transduced into different *S. aureus* backgrounds: AH1263 (blue), NCTC-8325 (red), TCH1516 (green), Newman (purple), JE2 (orange), COL (black), Mu50 (brown), and UAMS-1 (dark blue). Strains were grown under similar conditions as in panel B. Promoter activities for Teg41 (**D**) and *psmα* (**E**) were calculated as described in Materials and Methods. Values were normalized to 100% for AH1263 ± SD. ****P < 0.0001 by analysis of variance corrected with Dunnett’s multiple comparison test.

The Teg41 and αPSM genes are genetically linked in staphylococci. The two genes are divergently transcribed and are only found in strains of *Staphylococcus aureus* and *Staphylococcus argenteus*, a closely related strain that until 2015 was classified as *S. aureus* clonal complex (11). Only 230 bp separates the predicted TSS of Teg41 from the start codon of *psmα1*, raising the question of whether Teg41 expression mirrors that of the *psmα* transcript. To investigate the expression of both Teg41 and *psmα* simultaneously, we constructed a dual fluorescence reporter plasmid pPRB4 (Fig. 1A) using the pMK4 backbone plasmid (12). A 268 bp region (encompassing the 230 bp of the *psmα*-Teg41 promoter region and an additional 38 bp downstream of the predicted Teg41 TSS) was cloned between the Superfolder GFP (sGFP) and mCherry coding sequences. Due to the absence of a ribosome binding site within the Teg41 promoter, the translational initiation region (TIR) from the pJB185 plasmid (13) was added before the start codon of mCherry (Fig. 1A). *S. aureus* wild-type (WT) strain AH1263 containing the dual fluorescent reporter plasmid was grown in tryptic soy broth (TSB) in a 96-well plate for 16 h, during which bacterial density (OD_600_) and fluorescence intensity for both GFP (λ488/507) and mCherry (λ587/610) were monitored as proxies for *psmα* and Teg41 promoter activity, respectively. The maximum fluorescence intensity for each promoter was set at 100% activity. The *psmα* promoter remained inactive during the first 5 h of growth but then sharply increased during the late-exponential phase (Fig. 1B). In contrast, the Teg41 promoter showed low expression during the first 2 h of growth, followed by a gradual increase over time (Fig. 1B). We also observed a small plateau phase for Teg41 promoter activity before the *psmα* promoter activates. To further analyze the expression patterns, we plotted the fluorescence intensity against OD_600_ (Fig. 1C). The *psmα* promoter was activated when cell density reached OD_600_ = 0.85, while the Teg41 promoter displayed relatively continuous activity throughout the growth period (Fig. 1C). These results suggest that, despite the small distance separating the *psmα* locus from Teg41, the two promoters exhibit distinct expression kinetics: (i) the *psmα* promoter has an inducible pattern dependent on cell density, and (ii) Teg41 promoter activity is relatively constant during growth in TSB. Next, we wanted to assess the expression of both Teg41 and *psmα* in different *S. aureus* backgrounds. The dual reporter plasmid was phage-transduced into *S. aureus* TCH1516 (clonal complex CC8, MRSA) (14), JE2 (plasmid-cured strain of AH1263, CC8, MRSA) (15), Newman (CC8, MRSA) (16), COL (CC8, MRSA) (17), Mu50 (CC5, MRSA/vancomycin-intermediate *S. aureus*) (18), UAMS-1 (CC30, methicillin-sensitive *Staphylococcus aureus* [MSSA]) (19), and NCTC-8325 (CC8, MSSA) (20). Fluorescence intensities and OD_600_ were monitored over time, and promoter activities were calculated as described in Materials and Methods to account for the different growth rates of each strain. Promoter activities were normalized to AH1263 values set at 100%. No difference in Teg41 promoter activity was observed in any of the strains tested (Fig. 1D). In contrast, for the *psmα* promoter, strain Newman showed a significant increase in activity (255% versus 115%) compared to AH1263 (Fig. 1E). No *psmα* promoter activity was detected for strains COL, Mu50, and UAMS-1. To validate these findings, we performed reverse transcription quantitative PCR (RT-qPCR) targeting Teg41 and *psmα* in the AH1263, Newman (high *psmα* expression), and UAMS-1 (low *psmα* expression) strains (Fig. S1). The strains were grown in a 96-well plate at 37°C in TSB, and RNA was isolated after 3 or 6 h of growth. Eight individual wells were pooled and centrifuged prior to processing for RNA extraction and RT-qPCR. Results closely mirrored the promoter activity recorded for the PSMα promoter at 3 h, with high levels of transcript in Newman and low levels of transcript in UAMS-1, which was also confirmed at 6 h. The RT-qPCR data indicated that lower levels of Teg41 RNA were observed in UAMS-1 at 3 and 6 h. The apparent lower Teg41 RNA transcript abundance in UAMS-1 could be the result of a higher RNA degradation in the strain. In conclusion, results outlined above demonstrate that Teg41 promoter activity and RNA levels remain largely consistent across different *S. aureus* strains, while *psmα* promoter activity and RNA abundance vary among them.

### Determination of minimal Teg41 promoter region and mapping extremities of Teg41 transcript

**Fig 2:**
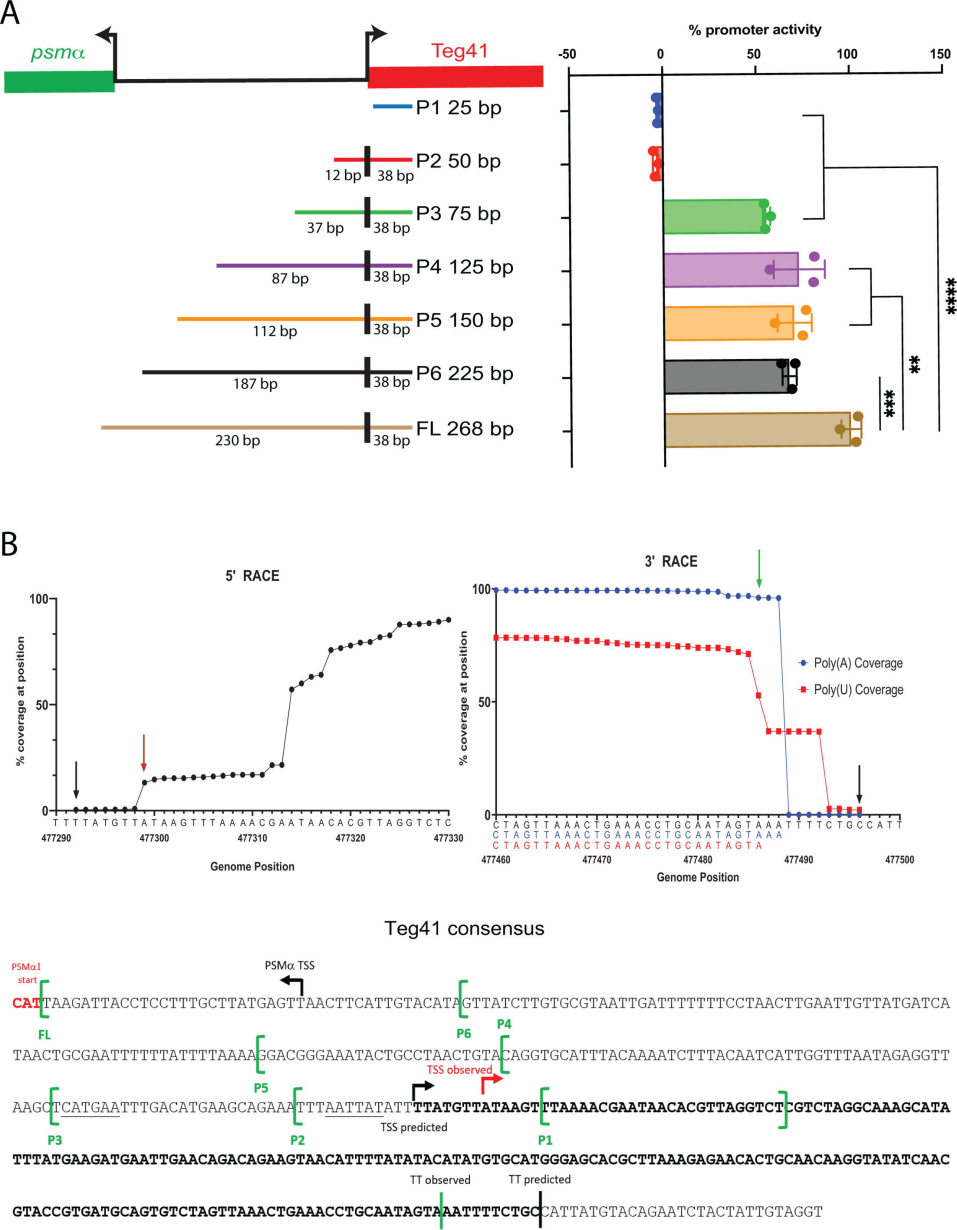
The Teg41 promoter is defined within a small region of 37 bp from the predicted TSS. (**A**) Effect of truncated Teg41 promoter on mCherry expression. Plasmid pPRB4 was used as a template to remove various regions of the Teg41 promoter (P1, full-length [FL]). Promoter activity is expressed as a percentage relative to the full-length promoter, which was set at 100% ± SD. **P < 0.01, ***P < 0.001, and ****P < 0.0001, as calculated by analysis of variance with Tukey’s multiple comparison test. (**B**) Identification of 5′ and 3′ ends of Teg41. Graphs depict mapping of reads recovered from nanopore cDNA sequencing reactions. Locations of previously predicted Teg41 5′ and 3′ ends are indicated by black arrows. Green arrows depict experimentally determined Teg41 5′ and 3′ ends. Based on the analysis of the reads obtained in the poly(A) and poly(U) reactions, the predicted 3′ end of Teg41 terminates on an adenine residue at position 477,486. Blue and red sequences shown for poly(A) and poly(U) reactions depict the consensus sequence for all reads in the sequencing reaction.

To further investigate which regions of the Teg41 promoter are necessary for expression, we constructed various truncated promoter sequences in the dual reporter plasmid pPRB4 using *in vivo* assembly. We analyzed the expression of seven different promoter lengths: P1 (25 bp, located at +13 bp relative to the predicted Teg41 TSS), P2 (50 bp, located at −12 bp), P3 (75 bp, located at −37 bp), P4 (125 bp, located at −62 bp), P5 (150 bp, located at −87 bp), P6 (225 bp, located at −162 bp), and the full-length (FL) native promoter (268 bp in pPRB4) (Fig. 2A). *S. aureus* WT strains carrying these constructs were grown in TSB, and promoter activities were assessed by measuring mCherry fluorescence normalized to OD_600_. The activities were then expressed as a percentage relative to the full-length promoter set at 100%. The truncated promoters P1 and P2 exhibited no activity, while promoters P3, P4, P5, and P6 showed similar activities, ranging from 50% to 75% of the full-length promoter activity. Only the full-length promoter (268 bp) displayed maximum activity. These results suggest that the proximal elements necessary for Teg41 promoter activity are located within the P2–P3 region, and potential distal elements are within the P6–full-length region (46 bp downstream of the *psma* start codon).  To investigate the extremities of the Teg41 transcript, we performed rapid amplification of cDNA ends (RACE) PCR. For the 5′ extremities, we employed a template-switching approach, while for the 3′ end, we used poly-adenylation and poly-uridylation coupled to reverse transcriptase (see Materials and Methods for details). Given the low GC% content of the *S. aureus* genome, we applied both poly-adenylation and poly-uridylation to ensure comprehensive mapping of the transcript’s terminus. This approach allowed us to capture multiple consecutive A or T residues at the 3′ end, ensuring that an A-tailed or U-tailed product would reliably align with the true sequence. Both final PCR products were sequenced using nanopore technology, allowing us to obtain reads that were subsequently mapped onto the *S. aureus* USA300 genome to identify transcript extremities (Fig. 2B). Determination of the 5′ end of Teg41 via template switching resulted in 18% of reads mapping to position 477,299, which is 7 nt downstream of the predicted Teg41 TSS. A sharp increase in coverage (60% of all reads) was observed at position 477,314, suggesting that either the 5′ end of Teg41 is processed and/or that Teg41 possesses multiple TSSs (i.e., positions 477,299 and 477,314). For the 3′ end, poly(A) PCR results showed a sharp drop in coverage at position 477,489, while poly-U PCR consensus coverage dropped at position 477,487. Collectively, these data suggest that the termination site of the Teg41 transcript is located at position 477,487, 9 nt upstream of the previously predicted termination site (Fig. 2B).

Based on the results of the 5′ RACE analysis, the TSS of Teg41 is not located within the P1 promoter fragment, and the P2 promoter fragment begins 7 nt upstream of the TSS. This likely explains why no transcriptional activity was observed from these constructs. The P3 promoter fragment, which does demonstrate transcriptional activity, contains 32 nt upstream of the Teg41 TSS, and potential −35 and −10 promoter elements are present within this sequence (underlined in Fig. 2B). Therefore, we hypothesize that this region contains the core Teg41 promoter.

### Identification of transcriptional factors of the Teg41 promoter

**Fig 3:**
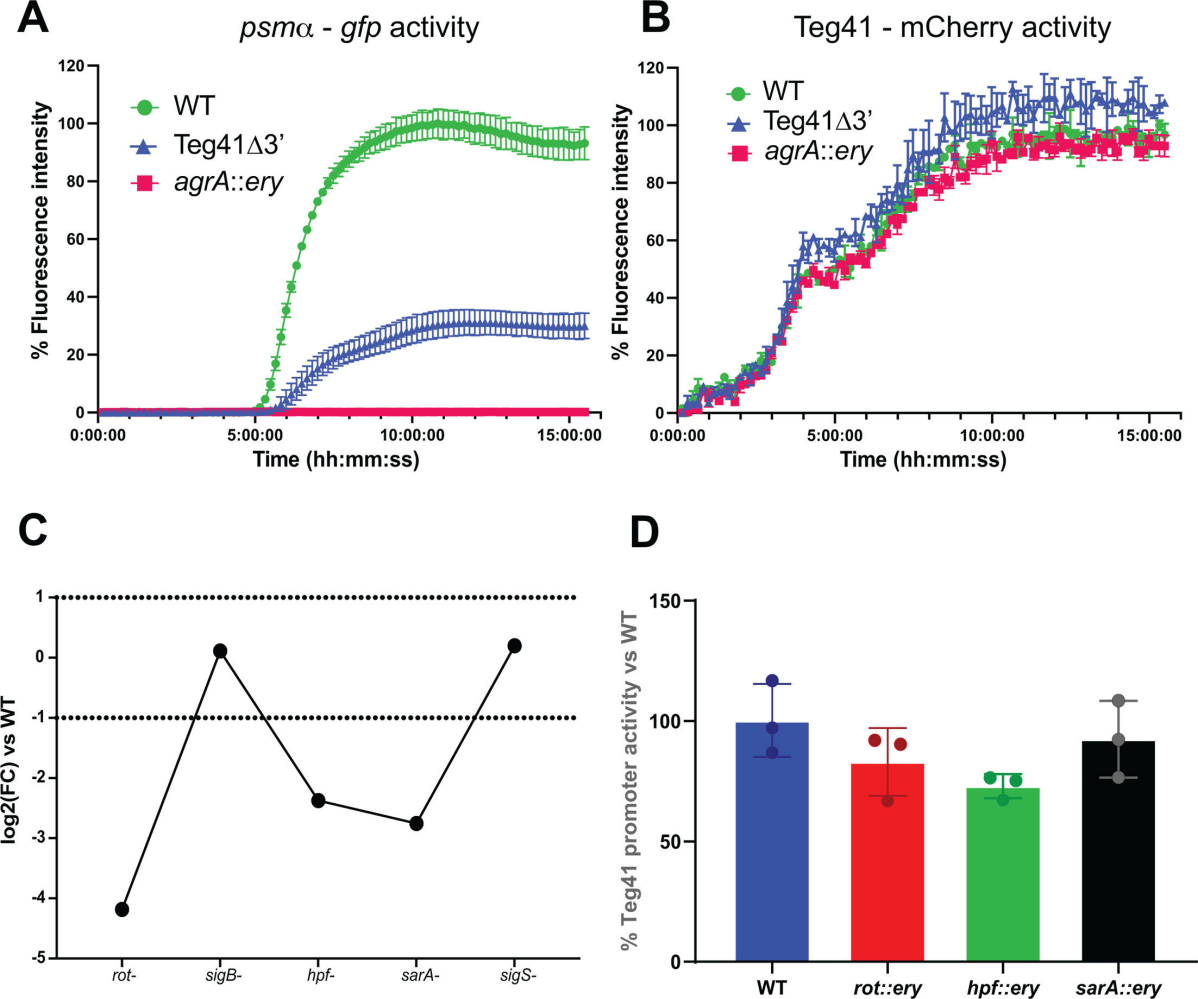
Teg41 promoter activity is relatively stable in the absence of global regulatory proteins. (**A and B**) Plasmid pPRB4 was phage-transduced into *S. aureus* AH1263 (WT), AH1263 Teg41Δ3′, or AH1263 *agrA*::ery strains, and the fluorescence intensities of sGFP (**A**) and mCherry (**B**) were recorded as proxies for *psmα* and Teg41 promoter activities, respectively. Fluorescence intensity is expressed as a percentage relative to the WT strain, which was set at 100%. (**C**) Teg41 expression in five transcriptional regulator mutants from RNA-seq. Results are shown as log2 fold change in expression compared to the WT strain in each experiment. Dotted lines represent log2FC >1 or <−1. (**D**) Plasmid pPRB4 was phage-transduced into *S. aureus* JE2 WT (blue), *rot*::ery (red), *hpf*::ery (green), or *sarA*::ery (black), and Teg41 promoter activity was monitored as previously described. Promoter activity is expressed as a percentage relative to the JE2 WT strain, which was set at 100% ± SD.

The results above suggest that the Teg41 promoter P6–full-length region may contain distal regulatory elements (due to the increased transcription observed in FL compared to P6). Since this region is close to the *psmα* locus and AgrA is a key regulator of *psmα* expression (21), we investigated the impact of AgrA on Teg41 expression. As expected, in an *agrA* mutant, there was no *psmα* promoter activity (Fig. 3A). However, there was no change in Teg41 promoter activity in the *agrA* mutant background compared to the wild-type strain (Fig. 3B), indicating that AgrA does not regulate Teg41 expression. We also explored whether Teg41 could autoregulate its own expression. While *psmα* promoter activity was reduced by 70% in the Teg41 mutant strain (Fig. 3A), confirming previous findings from our group (10), there was no difference in Teg41 promoter activity in the Teg41 mutant strain compared to the wild-type strain (Fig. 3B), indicating that Teg41 does not autoregulate its own expression. Next, we mined RNA-seq data available from public data sets to investigate potential transcriptional regulators involved in Teg41 expression (Table S1). We first analyzed the impact of each two-component system (TCS) on Teg41 expression. We used data generated by phosphomimetic response regulators to determine whether activation of individual TCS would modulate Teg41 expression (22). Our analysis revealed that Teg41 expression was unaltered following expression of the active form of each *S. aureus* response regulator (Fig. S2). We continued our analysis by investigating Teg41 expression in RNA-seq data publicly available for transcriptional factor mutants (Table S1). We analyzed data for the repressor of toxins *rot*, alternative sigma factors *sigB* and *sigS*, hibernation factor *hpf*, and staphylococcal accessory regulator *sarA* and compared mutants to the wild-type strains in each condition for Teg41 expression (Fig. 3C). Analysis showed that Teg41 expression was repressed in the *rot* (log2 fold change [log2FC] = −4.2), *hpf* (log2FC = −2.4), and *sarA* (log2FC = −2.8) mutants (Fig. 3C). To confirm these results, we monitored both the *psmα* and Teg41 promoters using the dual fluorescence reporter in the appropriate mutant backgrounds. We used mutants available from the Nebraska Transposon Library and compared results to the wild-type JE2 strain (15). Only inactivation of *hpf* reduced the activity of the *psmα* promoter to about 60% of the WT strains, although not statistically significant (*P* value = 0.0862) (Fig. 3D). No difference in Teg41 promoter activity was observed for any mutants tested. These results suggest that Teg41 transcription is not controlled by the transcriptional factors tested in our conditions, and the decrease in Teg41 RNA levels observed by RNAseq may be due to altered rates of degradation in these backgrounds.

### Response of Teg41 and *psmα* promoters to environmental stimuli found during infection

**Fig 4:**
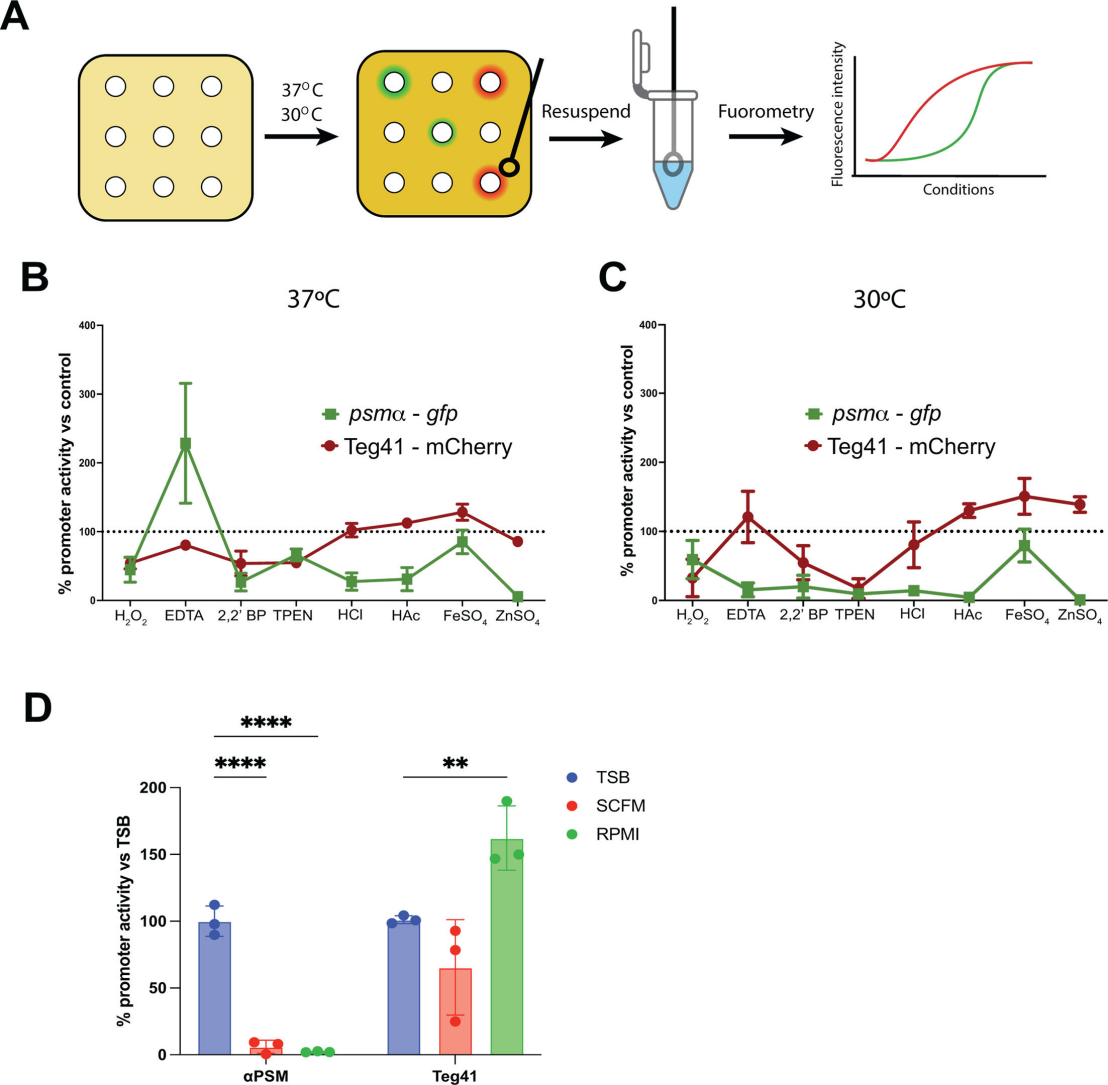
Environmental stimuli influence the promoter activities of Teg41 and *psmα*. (**A**) Schematic of the fluorescence disk assay. *S. aureus* WT carrying the pPRB4 plasmid is spread onto tryptic soy agar supplemented with chloramphenicol (10 µg/mL). After the plates are dried, disks soaked in different stimuli are placed onto the agar and incubated overnight at 37°C or 30°C. Bacteria are then collected either from the area of disk contact (if no inhibition is observed) or from the edge of the inhibition zone, resuspended in 1× phosphate-buffered saline, and the fluorescence intensity and OD_600_ (used to normalize bacterial density) are measured using a plate reader. (**B and C**) Fluorescence disk assay conducted at 37°C (**B**) or 30°C (**C**) in the presence of H_2_O_2_ (33%), EDTA (0.5 M), 2,2′-bipyridyl (2,2 BP, 64 mM), N,N,N′,N′-tetrakis(2-pyridinylmethyl)-1,2-ethanediamine (TPEN; 40 mM), hydrochloric acid (HCl; 1 M), acetic acid (HAc; 1 M), or supplements of iron sulfate (FeSO_4_) or zinc sulfate (ZnSO_4_; 100 mM). Results are shown as promoter activity expressed as a percentage ± SD relative to the control condition (disk soaked in water), which was set at 100%. (**D**) Teg41 and *psmα* promoter activities in nutrient-limited media: synthetic cystic fibrosis medium (SCFM; red) or Roswell Park Memorial Institute (RPMI; green). Results are shown as a percentage of promoter activity ± SD relative to the control condition (TSB; blue), which was set at 100%. **P < 0.01, ****P < 0.001, as calculated by two-way analysis of variance and corrected by Dunnett’s multiple comparison test.

Next, we wanted to assess Teg41 and *psmα* promoter activity under different stimuli found during infection. We first assessed Teg41 expression in publicly available RNA-seq data (Table S1) and compared results to the control condition in each experiment. *S. aureus* grown in the presence of calprotectin (log2FC = −2.1) and urine (log2FC = −2.0) showed reduced Teg41 expression (Fig. S3). To confirm and expand the conditions that modulate Teg41 promoter activity, we performed a fluorescent disk diffusion assay (Fig. 4A). Briefly, a lawn of *S. aureus* WT carrying the dual fluorescent reporter pPRB4 was grown on tryptic soy agar (TSA) + Cm10. Disks soaked in different stimuli [H_2_O_2_ 33% for oxidative stress, EDTA 0.5 M as a global metal chelator, 2,2′-bipyridyl 64 mM as an iron-specific chelator, N,N,N′,N′-tetrakis(2-pyridinylmethyl)-1,2-ethanediamine (TPEN) 40 mM as a zinc-specific chelator, hydrochloric acid HCl 1 M, acetic acid HAc 1 M, supplements of iron sulfate FeSO_4_ or zinc sulfate ZnSO_4_ 100 mM, and water for control] were then applied onto plates, which were incubated at 30°C or 37°C to mimic temperatures found during nasal colonization or infection, respectively (23, 24). After 24 h, GFP and mCherry fluorescence intensity were measured and normalized to OD_600_ (see Materials and Methods for additional details). At 37°C, Teg41 promoter activity was reduced in the presence of H_2_O_2_ (54.3% of control), EDTA (80.4%), 2,2′-bipyridyl (53.8%), TPEN (54.9%), and ZnSO_4_ (85.6%). The stronger repression of Teg41 expression by 2,2′-bipyridyl (iron chelator) and TPEN (zinc chelator), compared to EDTA (broad-spectrum metal chelator), suggests that Teg41 is more sensitive to iron and zinc limitation than to manganese depletion.

However, Teg41 promoter activity was slightly stimulated in the presence of HAc (112.4%) and FeSO_4_ (128.4%) (Fig. 4B). The *psmα* promoter showed a different pattern of expression. H_2_O_2_, 2,2′-bipyridyl, and TPEN reduced *psmα* promoter activity (44.7%, 26.7%, and 66.1%, respectively). Similar results were observed for the Teg41 promoter under these conditions. In contrast, the presence of EDTA sharply increased the activity (228.4%) of *psmα* promoter, and the presence of ZnSO_4_ sharply decreased *psmα* promoter activity (5.5%). Notably, the presence of HCl and HAc decreased *psmα* promoter activity (27.4% and 30.9%, respectively), which is the opposite to the Teg41 promoter response for HAc (Fig. 4B). At 30°C, Teg41 promoter activity was similar to the activity observed at 37°C (Fig. 4C). Interestingly, at 30°C, the psmα promoter exhibited a similar activity pattern to that observed at 37°C, except in the presence of EDTA, which caused a sharp decrease in activity (15.4%). This condition also showed greater variability compared to other stimuli (Fig. 4C). Together, these results suggest that (i) the Teg41 promoter activity does not respond to temperature changes; (ii) the Teg41 promoter displays variations of activity in response to the environmental stimuli tested; and (iii) the *psmα* promoter has a different response to EDTA, depending on the growth temperature.

Finally, we assessed the promoter activity of both *psmα* and Teg41 in liquid culture using physiologically relevant media. We used synthetic cystic fibrosis medium (SCFM) and Roswell Park Memorial Institute (RPMI) medium, which have been previously used to mimic low-nutrient environments found in cystic fibrosis and human plasma, respectively (25–27). *S. aureus* was grown in TSB, SCFM, or RPMI for 16 h at 37°C, and GFP and mCherry fluorescence intensity and bacterial density (OD_600_) were measured. Promoter activity was calculated as above and compared to the activity in TSB, which was set at 100% (Fig. 4D). Interestingly, *psmα* promoter activity was significantly reduced in SCFM (10.2% of the activity in TSB) and RPMI (3.1%), while Teg41 promoter activity was not significantly changed in SCFM and was increased in RPMI (155.3%) (Fig. 4D). We also plotted the fluorescence intensity against OD_600_, as outlined above for TSB (Fig. 1C). The activities of the Teg41 and *psmα* promoters display a linear correlation with OD_600_ in SCFM, indicating that the activity of both promoters seems to be dependent on growth. In RPMI, the *psmα* promoter shows no relationship with bacterial growth, but Teg41 promoter activity still correlates with OD_600_ (Fig. S4). In conclusion, Teg41 promoter activity, while relatively constant, can display some variation in response to environmental stimuli found during infection. Teg41 promoter activity is stable or increased in nutrient-limited conditions (unlike the *psmα* promoter), and Teg41 promoter activity correlates with bacterial growth in limited nutrient media, as seen for TSB.

### *S. aureus* displays different subpopulations of cells expressing varying levels of Teg41 and *psmα* in TSB and RPMI

**Fig 5:**
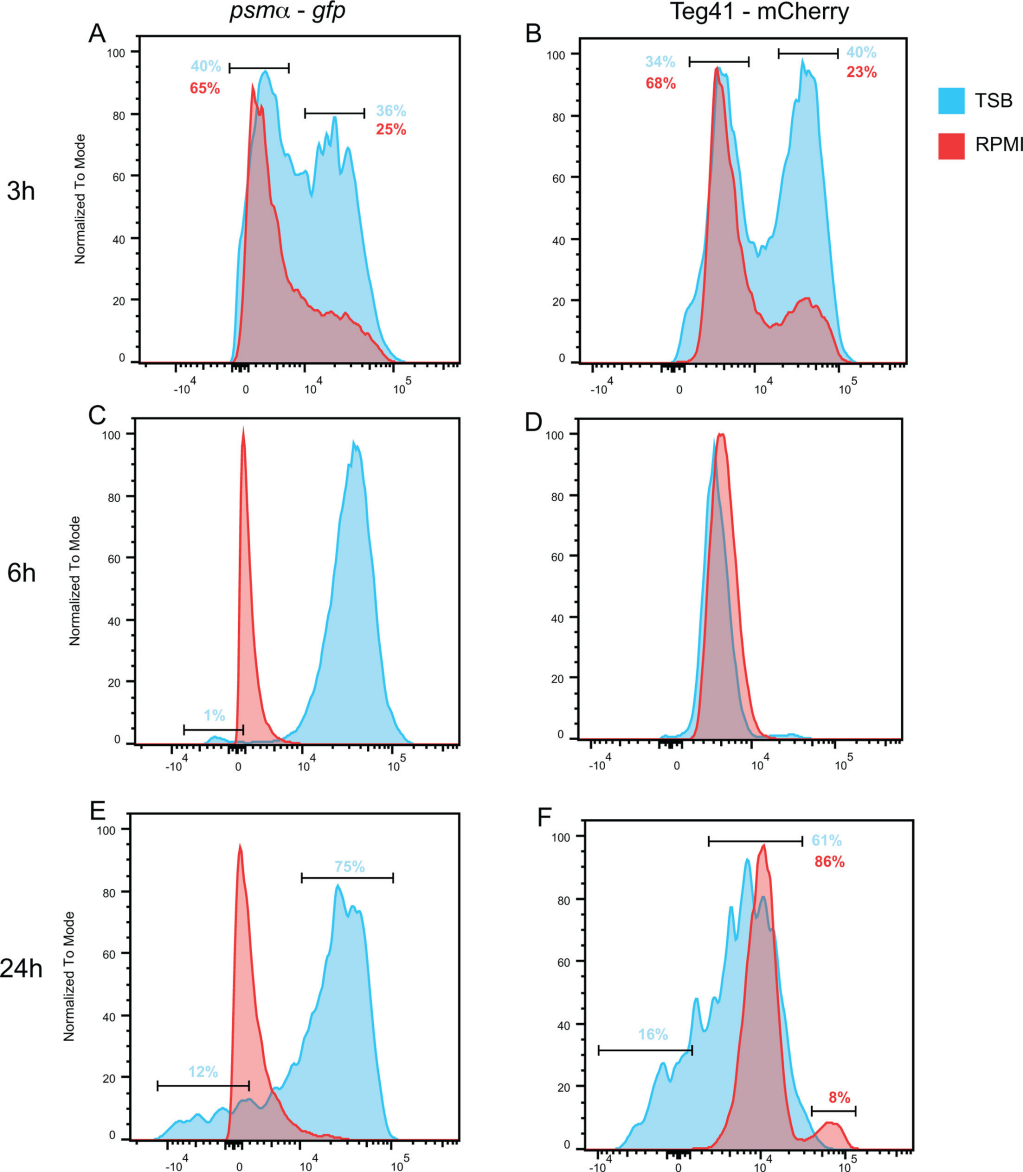
Flow cytometry analysis reveals heterogeneous expression of Teg41 and *psmα. S. aureus* wild-type (WT) carrying pPRB4 was grown in TSB (blue) or RPMI (red) for 3 h (**A **and **B**), 6 h (**C **and **D**), or 24 h (**E** and **F**) before assessing the expression of Teg41 (mCherry) and *psmα* (GFP) by flow cytometry. Modal normalization was applied per condition, aligning the dominant fluorescence peak (mode) of GFP or mCherry to 100%. Subpopulations of interest are highlighted within intervals, with the percentage of the total population shown for each condition.

Growth in RPMI was one of the few conditions where the Teg41 promoter showed increased activity compared to TSB; therefore, we further analyzed the dynamics of Teg41 and *psmα* promoter expression in RPMI at the single-cell level using quantitative flow cytometry analysis (Fig. 5). *S. aureus* WT carrying the dual fluorescent reporter was grown in flasks in either TSB or RPMI, and Teg41 and *psmα* promoter activities were measured by flow cytometry at 3 h (log phase), 6 h (late log phase), and 24 h (late stationary phase) of growth. At 3 h in RPMI and TSB, Teg41 and *psmα* promoter activities were not homogeneous (Fig. 5A and B). *S. aureus* cells were clustered into two different populations: lower *psmα* and Teg41 promoter activities (in TSB, 40% and 34% of cells, respectively; in RPMI, 65% and 68% of cells, respectively) and higher *psmα* and Teg41 promoter activities (in TSB, 36% and 25%, respectively; in RPMI, 40% and 23%, respectively). Interestingly, at 6 h, the activities of both *psmα* and Teg41 promoters were homogeneous in TSB and RPMI, with higher *psmα* promoter activity in TSB compared to RPMI and higher Teg41 promoter activity in RPMI (Fig. 5C and D). Notably, a small subpopulation (1%) exhibited no *psmα* promoter activity in TSB (Fig. 5C). *S. aureus* grown for 24 h showed heterogeneity in promoter activity in both TSB and RPMI (Fig. 5E and F). At 24 h the *psmα* promoter showed little to no activity in RPMI (Fig. 5E), but *S. aureus* displayed two different populations in TSB: 75% of cells expressed high levels of *psmα*, and 12% showed low/no expression of *psmα*. For the Teg41 promoter, cells grown in TSB showed a wide range of activity, with most cells (61%) displaying high activity and 16% of cells showing low to no Teg41 activity (Fig. 5F). In RPMI, the Teg41 promoter exhibited higher activity than in TSB, with most cells (86%) showing higher activity than the maximum observed in TSB. Interestingly, a small subset of cells (8%) exhibited very high Teg41 activity (about 8- to 10-fold more than the majority of the population) (Fig. 5F). These results demonstrate a few important findings. First, Teg41 and *psmα* expressions are not similar during growth in TSB and RPMI. While *psmα* is highly expressed in TSB during stationary phase, expression from this promoter is almost completely repressed in RPMI at equivalent time points. Differences in Teg41 expression during growth in TSB and RPMI were also observed at early (3 h) and late (24 h) time points. Second, these results clearly show that Teg41 and *psmα* expressions are not homogeneous throughout the population. Rather, subpopulations exist within the bacterial cultures that display differential levels of gene expression. Notably, when comparing population overlap, we consistently observed that cells with higher Teg41 expression also exhibited elevated levels of *psmα*, whereas cells with lower Teg41 expression showed reduced *psmα* levels. This pattern supports the role of Teg41 as a potential activator of *psmα* expression (Fig. S5). This finding has important consequences for the study of bacterial gene expression and further highlights the need to study gene expression in individual cells.

### Addition of transition metals modifies subpopulation dynamics observed for Teg41 and *psmα* promoters in RPMI

**Fig 6:**
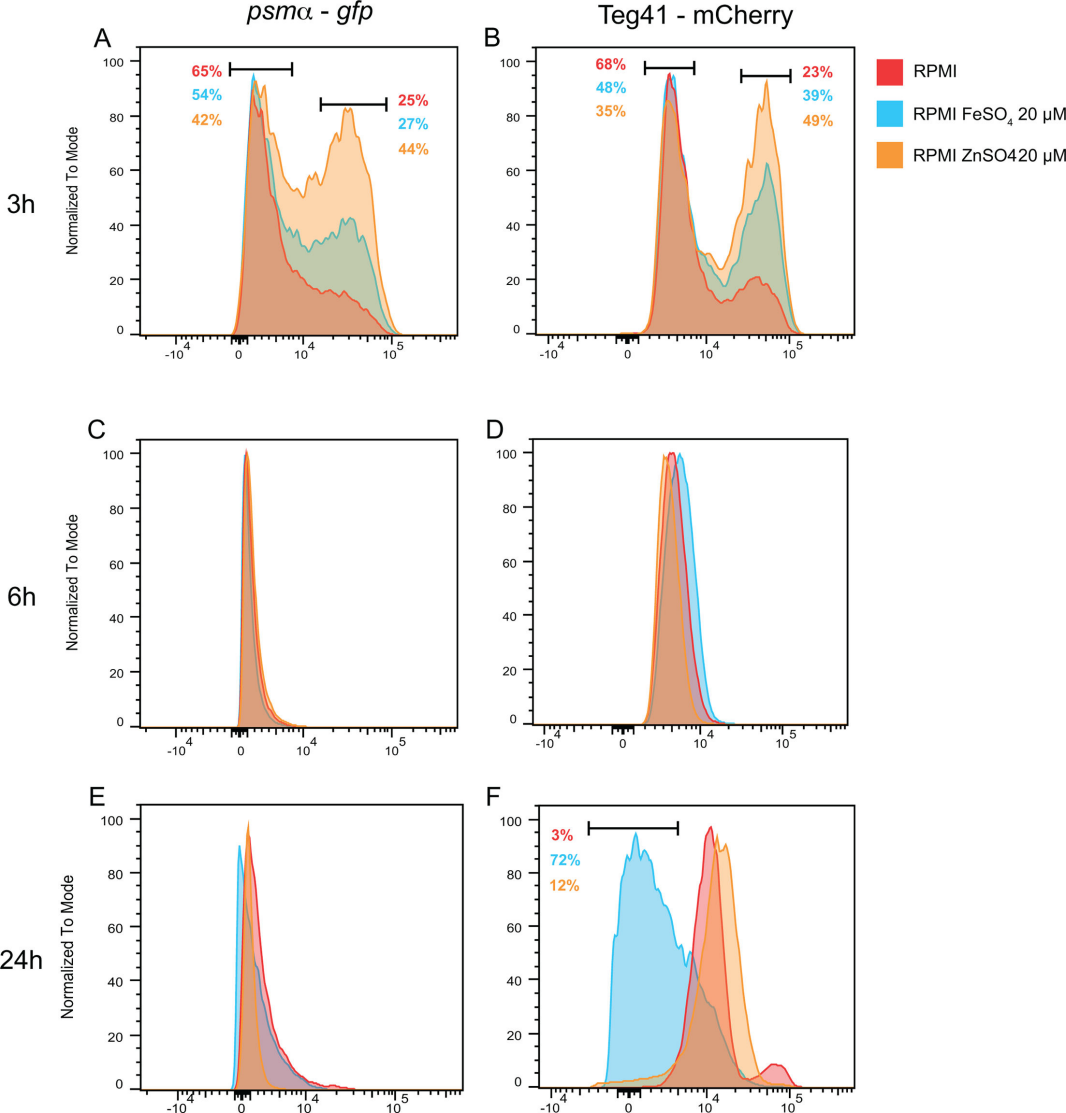
The addition of transition metals alters the proportion of subpopulations expressing Teg41 and *psmα. S. aureus* wild-type (WT) carrying pPRB4 was grown in RPMI (red) supplemented with 20 µM of either FeSO_4_ (blue) or ZnSO_4_ (orange) for 3 h (**A** and **B**), 6 h (**C** and **D**), or 24 h (**E** and **F**) before assessing Teg41 (mCherry) and *psmα* (GFP) expression by flow cytometry. Modal normalization was applied per condition, aligning the dominant fluorescence peak (mode) of GFP or mCherry to 100%. Subpopulations of interest are highlighted within intervals, with the percentage of the total population shown for each condition.

RPMI is considered a nutrient-limited medium compared to TSB, notably for transition metal availability (iron and zinc). In RPMI, the concentrations of zinc and iron are trace compared to the high levels found in TSB (around 20 µM of iron and 8 µM of zinc) (28, 29). Moreover, disk diffusion assays showed that depletion of iron/zinc changed Teg41 and *psmα* promoter activity dynamics (Fig. 4B). Thus, we investigated whether the addition of iron and zinc to RPMI could influence Teg41 and *psmα* expressions and/or modify the observed subpopulations. *S. aureus* was grown in RPMI in the presence of zinc or iron (20 µM), and promoter activities were monitored at 3, 6, and 24 h by flow cytometry and expression levels compared to *S. aureus* grown in RPMI alone. For the *psmα* promoter, the addition of zinc increased the percentage of cells with high promoter activity at 3 h (from 25% in RPMI to 44% in RPMI + zinc), but transition metals had no impact at later time points (Fig. 6A, C, and E). For the Teg41 promoter, at 3 h, the addition of zinc or iron similarly increased the percentage of cells with high activity, as observed with the *psmα* promoter (Fig. 6B). Addition of iron and particularly zinc led to similar Teg41 expression subpopulations as those observed for *S. aureus* grown in TSB (Fig. 5B). At 6 h, no difference was seen following the addition of zinc or iron, with a homogeneous population displaying a similar level of Teg41 promoter activity (Fig. 6D). Remarkably, at 24 h, the presence of iron reduced Teg41 promoter activity (72% of cells with low levels of Teg41 expression) compared to RPMI or RPMI + zinc, which had similar levels of Teg41 promoter activity (Fig. 6F). Collectively, these data show that the presence of transition metals impacts the dynamics at the single-cell level of Teg41 and *psmα* promoter activities in RPMI, with iron and zinc increasing activities at the early phase of growth for both promoters and iron negatively impacting Teg41 promoter activity during late stationary growth.

## DISCUSSION

We have previously shown that Teg41 is a crucial sRNA for *S. aureus* virulence, as it controls the expression of numerous virulence factors, including the PSMα peptides (9, 10). The small distance separating the *psmα* locus from Teg41 prompted us to investigate the dynamic expression of this region. For the first time in *S. aureus*, we used a dual fluorescent reporter to study the relationship between the divergent *psmα* and Teg41 promoters. Our data demonstrate that Teg41 has its own unique promoter, which can respond to various stimuli encountered during infection in a heterogeneous manner, with cells displaying different levels of promoter activity, depending on the conditions. In TSB, the Teg41 promoter displays constitutive expression, with activity that correlates with cell growth. In contrast, the *psmα* promoter expression is initiated only when cells reach quorum, as it has been described before (21). The distinct expression patterns of these two promoters are particularly interesting, given the small distance (203 bp) separating their respective TSS. In RPMI and SCFM, the *psmα* promoter is inactive, whereas the Teg41 promoter is expressed, with an activity level higher in RPMI than in TSB. This difference is notable because RPMI and SCFM are nutrient-limited media compared to TSB, containing lower concentrations of metals, vitamins, and amino acids (RPMI) or glucose (SCFM) (27), suggesting that the Teg41 promoter is not adversely affected by low nutrient availability, in contrast to the *psmα* promoter, and may in fact be enhanced by nutrient limitation. The increased activity of the Teg41 promoter in RPMI is intriguing and suggests that its role in *S. aureus* physiology could differ, depending on the availability of nutrients. RPMI is often used in studies because it mimics human plasma (25, 26). Our results suggest that *psmα* is not expressed—or would be minimally expressed—in plasma-like conditions, although this requires further clarification. These findings highlight a potential key role for Teg41 in this environment. These results corroborate our previous findings (10) that Teg41 has a role in *S. aureus* separate from that of regulating PSMα production. Further RNA-seq and proteomics studies are needed to investigate the impact of Teg41 in RPMI and during plasma exposure. In our investigation of the minimal promoter region responsible for Teg41 expression, we determined that the proximal element of the Teg41 promoter is located within 37 bp upstream of the TSS, as identified by RACE PCR. The first 15 bp of this region includes a high A/T sequence (5′-TAATTATATT-3′) that contains a putative −10 element (underlined; Fig. 2B). Between positions −37 and −25 (5′-TCATGAATTTGA-3′), we identified a putative −35 element (underlined). Interestingly, no additional promoter activity was observed when extending the sequence up to position −162, suggesting that under our experimental conditions, no additional regulatory elements are present in this region. Therefore, the correlation between Teg41 promoter activity and cell growth in different media appears to rely solely on the first 37 bp, containing the −10 and −35 elements, suggesting that the Teg41 promoter is relatively constitutive in *S. aureus*.

Although the proximal region of the Teg41 promoter (up to −187 nt) does not appear to influence activity, the distal region does seem to be important. The difference between the P6 promoter fragment and the full-length promoter is 43 bp. This region includes an AgrA binding site (necessary for *psmα* expression), which is cut in half in P6. Interestingly, as shown in Fig. 3, absence of AgrA does not affect Teg41 promoter activity, ruling out regulation by AgrA. This 43 bp region also encompasses the TSS of the *psmα* operon. It is possible that transcriptional activity from the *psmα* promoter may influence Teg41 promoter activity. We observed a small plateau phase in Teg41 activity just before quorum sensing is reached. When the *psmα* promoter is activated, Teg41 promoter activity resumes (Fig. 1). It is known that DNA topology can affect the transcription of divergent promoters. Transcription generates significant torsion in DNA during the elongation step by RNA polymerases, which can spread and influence neighboring promoters through a mechanism called transcription-supercoiling coupling (TSC) (30, 31). Studies by Sobetzko have shown that in configurations with two divergent promoters, where one is inducible, the activity of the non-inducible promoter can also increase upon induction of the inducible one (31, 32). In our case, when quorum is reached, the *psmα* promoter is induced, and we hypothesize that TSC stimulates Teg41 promoter activity. This creates a positive feedback loop whereby expression of the *psmα* operon stimulates Teg41 expression through TSC.

We have identified several stimuli that can modulate both the *psmα* and Teg41 promoter activities, which confirm studies described by others (33). Interestingly, while Teg41 promoter activity responds similarly at both 37°C and 30°C with small variation (50%–125% of activity, depending on stimuli and temperature), the *psmα* promoter showed significant temperature-dependent variation in activity. At 37°C, the *psmα* promoter is strongly stimulated by EDTA, a broad-spectrum metal chelator (calcium, manganese, iron, lead, zinc, and copper). However, at 30°C, EDTA represses *psmα* promoter activity. This suggests that temperature plays a crucial role in regulating *psmα* expression under different stimuli. It is well established that the limited availability of transition metals during nutritional immunity serves as a cue for bacteria to activate genes involved in invasion (34). However, we demonstrate here that the *psmα* promoter is specifically stimulated at 37°C by EDTA. When specific chelators like 2,2′-bipyridine (2,2′-BP) for iron or TPEN for zinc are used, the *psmα* promoter is repressed. This indicates that *psmα* expression might be stimulated when multiple transition metals are simultaneously deficient or when another metal ion—such as calcium, magnesium, or copper, which EDTA also targets—is absent. Because Teg41 promoter activity seems to be relatively constant, the variation of activity seen could be due to global physiological impact on cellular processes that might impact transcription and translation at the cell level or due to TSC of the *psmα* promoter. These findings lead us to speculate that in a commensal state, such as on the skin (30°C), *S. aureus* does not upregulate *psmα* expression in response to metal chelation. However, as *S. aureus* transitions to a pathogenic state and invades deeper tissues, the increase in temperature combined with the limited availability of various transition metals could trigger *psmα* expression. This hypothesis is consistent with previous data from our lab showing that PSM*α* production in *S. aureus* is temperature dependent, with higher temperatures giving rise to increased PSM*α* production (23).

We leveraged flow cytometry to analyze *psmα* and Teg41 promoter activity at the single-cell level. Our study reveals that the expression of *psmα* and *Teg41* is not homogeneous within the population, showing variability specifically at 3 and 24 h of growth. Notably, at 6 h, corresponding to the late-exponential growth phase in both TSB and RPMI, cells exhibited homogeneous expression of both promoters. This finding is significant, as it highlights that the most variation occurs outside the exponential growth phase. During the exponential phase, cells are actively replicating, and quorum-sensing mechanisms activate the *agr* system and RNAIII, leading to the regulation of about 15% of *S. aureus* genes (33, 35). A dogma shared in the community is that cell populations should display homogeneous expression during the exponential phase, and as cells exit the exponential phase, they become desynchronized. Interestingly, our data contradict this hypothesis at 3 h (log phase). We observed heterogeneous expression of both Teg41 and *psmα* with a subpopulation of cells with high Teg41 and *psmα* promoter activity, particularly in TSB. This distinct subpopulation reappears at 24 h for Teg41 but not for *psmα*.

Conlon et al. (36) demonstrated that during the stationary phase, persister cells—a small subpopulation in a dormant state that are tolerant to antibiotics—emerge due to reduced intracellular ATP levels. Furthermore, the inactivation of the TCA cycle can also trigger the formation of persister cells, independent of ATP levels (37). In the stationary phase, as nutrients become scarce and cells enter starvation mode, stress responses are activated; the TCA cycle activity is reduced; and the intracellular ATP level drops. It is possible that one subset of the population observed in our study during the stationary phase consists of persister cells. Interestingly, supplementing RPMI medium with either iron or zinc increases the proportion of cells expressing high levels of *psmα* and Teg41 at 3 h.

However, it remains unclear how these transition metals influence the dynamics of subpopulations, as their effects are not observed at later time points. Notably, cells grown in RPMI supplemented with 20 µM of ZnSO_4_ exhibit behavior remarkably similar to those in TSB, with comparable population percentages at 3 h. It is possible that the addition of transition metals in RPMI supports essential cellular processes, such as the proper incorporation of metals into metalloenzymes, enhancing their functionality. This, in turn, might contribute to the distinct subpopulations observed in our experiments. Nevertheless, further investigation is required to fully understand and characterize these populations.

In conclusion, our study reveals that Teg41 expression is relatively stable across various conditions, while *psmα* expression is highly sensitive to environmental stimuli, especially at 37°C and in the presence of EDTA. This indicates a regulatory mechanism that adapts *S. aureus* from a commensal to a pathogenic state. The observed variability in promoter activity at the single-cell level, particularly with transition metals, highlights the dynamic nature of these regulatory elements and suggests further investigation is needed to fully understand these mechanisms.

## MATERIALS AND METHODS

### Strains, plasmids, and growth conditions

Strains, plasmids, and oligonucleotides used in this study are provided in Tables 1 to 3, respectively. Strains carrying pPRB4 and its derivatives were plated onto TSA with chloramphenicol (10 µg/mL, Cm10). A single isolated colony was grown in 5 mL of TSB + Cm10 (Difco) or in RPMI medium 1640 (Difco) at 37°C with shaking at 250 rpm in glass tubes overnight. For experiments in flasks or 96-well plates, 1 mL of cells was pelleted and washed once with an equal volume of phosphate-buffered saline (PBS) at room temperature. Cell density was measured with a spectrophotometer at OD_600_. Flasks (12.5 mL) or 96-well plates (200 µL) containing either TSB, RPMI, RPMI + 20 µM FeSO_4_, 20 µM ZnSO_4_, or SCFM supplemented with Cm10 were inoculated with *S. aureus* cells to a final OD_600_ of 0.05. Cultures were incubated at 37°C at 250 rpm for 24 h (flasks) or at 37°C with double orbital shaking for 24 h (96-well plates) in a plate reader (H1 plate reader, Synergy; Agilent).

**TABLE 1:** Strains used in this study

Strain	Strain details	Description	Reference
PRB7	AH1263 + pPRB7_EV	*S. aureus* wild type with promotorless dual reporter plasmid	This study
PRB4	AH1263 + pPRB4	*S. aureus* wild type with dual reporter plasmid	This study
PRB2	AH1263 Teg41∆3′ + pPRB4	*S. aureus* Teg41 20 nt deletion at the 3′ end with dual reporter plasmid	(9)
PRB09	IM08B	Wild-type *Escherichia coli*	(38)
PRB10	AH1263 agrB::tn + pPRB4	*S. aureus* transposon mutants in *agrB* with dual reporter plasmid	This study
PRB30	AH1263 + pPRB30	*S. aureus* wild type with truncated promoter (25 bp)	This study
PRB31	AH1263 + pPRB31	*S. aureus* wild type with truncated promoter (50 bp)	This study
PRB32	AH1263 + pPRB32	*S. aureus* wild type with truncated promoter (75 bp)	This study
PRB33	AH1263 + pPRB33	*S. aureus* wild type with truncated promoter (150 bp)	This study
PRB48	AH1263 + pPRB48	*S. aureus* wild type with truncated promoter (225 bp)	This study
PRB49	AH1263 + pPRB49	*S. aureus* wild type with truncated promoter (125 bp)	This study
PRB60	JE2 + pPRB4	*S. aureus* wild type JE2 with dual reporter plasmid	This study
PRB61	JE2 rot::tn + pPRB4	*S. aureus* JE2 transposon mutant in rot with dual reporter plasmid	This study
PRB62	JE2 hpf::tn + pPRB4	*S. aureus* JE2 transposon mutant in hpf with dual reporter plasmid	This study
PRB63	JE2 srrB::tn + pPRB4	*S. aureus* JE2 transposon mutant in srrB with dual reporter plasmid	This study
PRB64	JE2 saeR::tn + pPRB4	*S. aureus* JE2 transposon mutant in saeR with dual reporter plasmid	This study
PRB65	JE2 sarA::tn + pPRB4	*S. aureus* JE2 transposon mutant in sarA with dual reporter plasmid	This study

**TABLE 2:** Plasmids used in this study

Plasmid	Plasmid details	Description	Reference
pPRB4	Dual reporter plasmid PSM*α*-Teg41	pMK4 backbone plasmid with full length PSM*α*-Teg41 promoter region inserted between *gfp* and *mCherry* genes	This study
pPRB7	Dual “reporter plasmid	pMK4 backbone plasmid with no promoter inserted between *gfp* and *mCherry* genes	This study
pPRB30	pPRB4-25 bp	Dual reporter plasmid with 25 bp of Teg41 region in frame with *mCherry*	This study
pPRB31	pPRB4-50 bp	Dual reporter plasmid with 50 bp of Teg41 region in frame with *mCherry*	This study
pPRB32	pPRB4-75 bp	Dual reporter plasmid with 75 bp of Teg41 region in frame with *mCherry*	This study
pPRB33	pPRB4-150 bp	Dual reporter plasmid with 150 bp of Teg41 region in frame with *mCherry*	This study
pPRB48	pPRB4-225 bp	Dual reporter plasmid with 225 bp of Teg41 region in frame with *mCherry*	This study
pPRB49	pPRB4-125 bp	Dual reporter plasmid with 125 bp of Teg41 region in frame with *mCherry*	This study

**TABLE 3:** Oligonucleotides used in this study^*a*^

Oligo name	Sequence	Description
OPRB9	atttgttgcatcaccttcaccttcacc	Forward screening pPB4
OPRB10	gtgtaccttcatatggacgaccttcacc	Reverse screening pPB4
OPRB31	ATGTCAAAAGGTGAAGAATTATTTACAG	Reverse pPB4 Teg41
OPRB32	aattcttcaccttttgacatTTAAAACGAATAACACGTTAGGTCT	Forward pPB4 Teg41 25 nt
OPRB33	aattcttcaccttttgacatTTTAATTATATTTTATGTTATAAGTTTAAAACGAATAACAC	Forward pPB4 Teg41 50 nt
OPRB34	aattcttcaccttttgacatTCATGAATTTGACATGAAGCAG	Forward pPB4 Teg41 75 nt
OPRB36	aattcttcaccttttgacatCAGGTGCATTTACAAAATCTTTACA	Forward pPB4 Teg41 125 nt
OPRB37	aattcttcaccttttgacatGGACGGGAAATACTGCC	Forward pPB4 Teg41 150 nt
OPRB40	aattcttcaccttttgacatGTTATCTTGTGCGTAATTGATTTTTTTC	Forward pPB4 Teg41 225 nt
OPRB17	ACTGGCCGTCGTTTTAC	Linearize pMK4 at M13 site forward
OPRB18	CATGGTCATAGCTGTTTCC	Linearize pMK4 at M13 site reverse
RKC45	GTAAAACGACGGCCAGTG	M13 forward
RKC46	GGAAACAGCTATGACCATG	M13 reverse
RKC1926	CACAGCATTTGGTACAG	Forward GyrB for qPCR
RKC1927	ATCGGCATCAGTCATAA	Reverse GyrB for qPCR
RKC1924	GTCTAGGCAAAGCATATTT	Forward Teg41 for qPCR
RKC1925	CTTGTTGCAGTGTTCTC	Reverse Teg41 for qPCR
RKC1939	CATCGCTGGCATCATTAAAG	Forward PSM*α* for qPCR
RKC1940	TTGCTACGAATTCCATGTGA	Reverse PSM*α* for qPCR
RKC2159	GCTAATCATTGCAAGCAGTGGTATCAACGCAGAGTACATrGrGrG	Template-switching oligo (5′ RACE)
RKC2160	TTGATATACCTTGTTGCAGT	Template-switching teg41 RT primer
RKC2161	AGCGTGCTCCCATGCACATATG	Template-switching teg41 gene-specific primer
RKC2162	CATTGCAAGCAGTGGTATCAAC	Template-switching oligo primer (5′ RACE)
RKC2268	GCGAGCACAGAATTAATACGACTCACTATAGGTTTTTTTTTTTTVN	3′ RACE RT adapter [to bind RNA poly(A) tails]
RKC2269	GCGAGCACAGAATTAATACGACTCACTATAGGAAAAAAAAAAAABN	3′ RACE RT adapter [to bind RNA poly(U) tails]
RKC2275	GCGAGCACAGAATTAATACGACT	3′ RACE outer primer
RKC2276	CGCGGATCCGAATTAATACGACTCACTATAGG	3′ RACE inner (nested) primer
RKC0333	GGTCTCGTCTAGGCAAAGCA	Teg41 3′ RACE outer
RKC2182	GgaattcGAAGATGAATTGAACAGACAG	Teg41 3′ RACE inner primer

^*a*^ RT, reverse transcription.

### Construction of dual reporter plasmid pPRB4 and derivatives

Superfolder GFP (sGFP), mCherry (codon optimized for *S. aureus*), and the promoter sequence of psmα-Teg41 (268 bp) were synthesized by Twist Bioscience. M13 forward and reverse sequences were added at the 5′ and 3′ ends, respectively, during synthesis. The TIR from the plasmid pJB185 (13) was also added before the mCherry start codon. The fragment was PCR amplified using oligos RKC45 and RKC46. Amplified fragments were inserted using the *in vivo* assembly method (39) into the plasmid pMK4 (12). Briefly, the plasmid pMK4 was linearized using inverse PCR and oligos OPRB17 and OPRB18. The plasmid and fragment (10 µL of each unpurified PCR product) were added to *Escherichia coli* IM08B competent cells (38). Cells were transformed, and transformants were selected on LB agar containing ampicillin (100 µg/mL). Clones were screened by PCR using oligos OPRB9 and OPRB10, and plasmids containing inserts were extracted using the QIAwave Plasmid Miniprep Kit (QIAGEN) prior to whole plasmid sequencing by Plasmidsaurus using Oxford Nanopore Technology with custom analysis and annotation. For truncated versions of the Teg41 promoter, the *in vivo* assembly strategy was selected (39) to remove parts of the Teg41 promoter using oligos OPRB31 paired with OPRB32 (25 bp), OPRB33 (50 bp), OPRB34 (75 bp), OPRB36 (125 bp), OPRB37 (175 bp), or OPRB40 (225 bp). Plasmids were transformed into *E. coli* IM08B, and plasmids were sequenced. Plasmids were then electroporated into *S. aureus* AH1263 (1 µg), and transformants were selected on TSA + Cm10.

### Determination of the 5′ and 3′ extremities of Teg41

To determine both 5′ and 3′ extremities of Teg41, two complementary approaches were used. For the 5′ end, template switching method, using the template switching reverse transcriptase enzyme mix (NEB M0466), was utilized according to the manufacturer’s instructions. Briefly, oligos RKC2160 and RKC2159 were used to perform the reverse transcription and template switching using DNA-free total RNA extracted from *S. aureus* grown at 6 h in TSB. Next, the 5′ region of Teg41 was amplified by high-fidelity Q5 PCR using oligos RKC2162 and RKC2161. Final products were purified and submitted for long-read nanopore DNA sequencing (Plasmidsaurus). For the 3′ extremity, total DNA-free RNA was either poly-adenylated or poly-uridylated using poly(A) polymerase (NEB M0276) or poly(U) polymerase (NEB M0337) according to the manufacturer’s protocol. Reactions were heat inactivated at 65°C for 20 min and used directly [20 µL for poly(A) and 25 µL for poly(U) reactions] as a template for reverse transcription using 1 µL of M-MulV RT (NEB M0253) and 1.45 µL of 3′ RACE adapter (10 mM) for poly(A) or 1.75 µL of 3′ RACE RT adapter (10 mM) for poly(U) tails. Then, two consecutive PCR reactions (outer PCR and inner PCR) were performed using Teg41-specific outer primer (RKC0333) and 3′ RACE Adapter Outer Primer (RKC2275) and Teg41-specific inner primer (RKC2182) and 3′ RACE Adapter inner primer (RKC2276). Final PCR products were checked on a 2% agarose gel for a single band and submitted for long-read nanopore DNA sequencing (Plasmidsaurus).

### Fluorometry

The growth of *S. aureus* was monitored in a plate reader (Synergy H1, Agilent) with the following parameters read every 10 min: OD_600_, fluorescence intensity for sGFP (λ488/507), and mCherry (λ587/610). To calculate the promoter activity and account for the growth rate of different mutants or growth rates in different media, the following equation (40) was used:


$$P=f_{ss}\;\times \;\mu \;\times \left(1\;+\;\frac{\mu }{m}\right),$$

where *P* is the promoter activity; *f*_ss_ is the slope of the tangent line through each point of the curve in a plot of fluorescence (F) as a function of OD; μ is the growth rate (min^−1^) during the exponential phase; and *m* is the ratio of ln (2) to the maturation constant for sGFP (39.1 min^−1^) and mCherry (81.4 min^−1^). Each promoter activity was then normalized to the wild-type strain AH1263 or to TSB conditions set at 100%.

### Fluorescence disk diffusion assay

Normalized overnight cultures (OD_600_ = 0.5) of *S. aureus* AH1263 wild type + pPRB4 were spread onto TSA + Cm10 with a sterile cotton swab on square petri dishes, and plates were dried for 15 min. Sterile disks (10 mm) were soaked in 10 µL of H_2_O_2_ (33%), metal chelator EDTA (0.5 M), iron chelator 2,2′-BP (40 mM), zinc chelator TPEN (40 mM), hydrochloric acid (HCl; 1 M), acetic acid (HAc; 1 M), iron sulfate (FeSO_4_; 100 mM), zinc sulfate (ZnSO_4_; 100 mM), or water as a control. Disks were then placed onto TSA plates and incubated at 30°C or 37°C overnight. The next day, a sterile loop was used to collect bacteria from the contact area of the disk or the edge of the inhibition halo (for H_2_O_2_, EDTA, 2,2′-BP, and TPEN). Bacteria were resuspended in a 1.5 mL Eppendorf tube with 200 µL of PBS (1×) and vortexed for 15 s. One hundred microliters was then transferred into a black 96-well plate, and OD_600_ and fluorescence intensity for sGFP (λ488/507) and mCherry (λ587/610) were measured. Promoter activity was calculated by dividing the fluorescence intensity for sGFP and mCherry by OD_600_.

### Reverse-transcription quantitative PCR

RNA was isolated from the wild-type strains using QIAGEN RNeasy Mini Kit. DNA was removed from the RNA samples with Turbo DNase (Thermo Fisher). Quantity and quality of the RNA were measured on an Agilent 2100 Bioanalyzer prokaryotic nano chip. RNA was then reversed transcribed using iScript cDNA synthesis kit (BioRad) to generate cDNA. gDNA isolated from AH1263 (non-DNeasy chromosomal DNA extraction from *S. aureus*) was diluted 200× and used as a positive control. Nuclease-free water was used as a negative control. Each well (96-well non-skirted qPCR plate, Bio-Rad) contains iTaq Universal Probes Supermix (#1725130, Bio-Rad), 500 nM F primer (Teg41 or PSMα), 500 nM R primer (Teg41 or PSMα), 250 nM F primer (*gyrB*), 250 nM R primer (*gyrB*), and nuclease-free water, along with 2.5 µL of 1:10 diluted cDNA. Adhesive film was applied to the plate to prevent evaporation (Bio-Rad). The plate was centrifuged for 1 min at 1,000 rpm, room temperature, in a centrifuge. CFX96 Touch Real-Time PCR Detection System (Bio-Rad) was used, along with CFX Maestro software. Data were then analyzed by setting relative expression to *gyrB* = 2^−(Cq_Teg41/PSMα_ − Cq^*^_gyrB_^*^)^.

### Flow cytometry assay and analysis

Flow cytometry was performed using a BD FACSMelody Cell Sorter (BD Biosciences) equipped with a 488 nm blue laser, a 640 nm red laser, and a 561 nm yellow-green laser. Fluorescein isothiocyanate (for GFP) and phycoerythrin (for mCherry) signals were detected using 527/32 and 613/18 filters, respectively. For 3 h cultures, 3 mL of *S. aureus* cells was pelleted at 10,000 × *g* for 1 min; the supernatant was discarded; and cells were resuspended in 1 mL of PBS (1×). For 6 and 24 h cultures, 1 mL of *S. aureus* cells was used and resuspended in 1 mL of PBS (1×). Data were acquired using BD FACSChorus software (BD Biosciences). A total of at least 10,000 events were collected per sample. Forward scatter (FSC) area and side scatter (SSC) area gates were set to exclude debris, and doublets were excluded based on SSC-width (W) versus SSC-height (H) and FSC-W versus FSC-H. Flow cytometry data were analyzed using FlowJo software (v.10.1, FlowJo LLC). GFP+ and mCherry+ *S. aureus* cells were gated based on the expression of GFP and mCherry markers.

### RNA-seq analysis

Accession numbers of RNA-seq data used in this study are listed in Table S1. Data were downloaded using the prefetch tool, and fastq files were extracted using fasterq-dump (SRA-toolkit, https://github.com/ncbi/sra-tools). Reads were trimmed (22 bp), and sequencing adapters were removed using trim-galore (v.0.6.10, https://github.com/FelixKrueger/TrimGalore). Reads were mapped against *S. aureus* rRNAs, and unmapped reads were mapped against *S. aureus* USA300 FPR3757 (updated for small RNA locations) (4) (CP000255.1) using bowtie2 (v.2.5.3) (41). Mapped reads were counted to each feature with R studio (v.4.3) (42) and the Subread package (v.2.0.6) with the featureCounts software (43). Differential expression analysis was performed using the DESeq2 package (44).

## SUPPLEMENTAL MATERIAL

Figure S1 msphere.00432-25-s0001.pdf Expression of PSMα and Teg41 transcripts in different *S. aureus* backgrounds.

Figure S2 msphere.00432-25-s0002.pdf Teg41 expression in phosphomimetic mutants analyzed by RNA-seq.

Figure S3 msphere.00432-25-s0003.pdf Teg41 expression across different stimuli.

Figure S4 msphere.00432-25-s0004.pdf Relationship between fluorescence intensity and bacterial growth for Teg41 and psmα promoters in SCFM and RPMI.

Figure S5 msphere.00432-25-s0005.pdf Coordinated expression of Teg41 and psmα in *S. aureus*.

Table S1 msphere.00432-25-s0006.xlsx Publicly available RNAseq data sets used in this study.

ASM does not own the copyrights to Supplemental Material that may be linked to, or accessed through, an article. The authors have granted ASM a non-exclusive, world-wide license to publish the Supplemental Material files. Please contact the corresponding author directly for reuse.
