# Supplementary material for: Analysis of Teg41 and PSMα promoter activity using a divergent fluorescent reporter plasmid
Source: mSphere. 2025 Oct 31;10(11):e00432-25. doi: 10.1128/msphere.00432-25 (PMC12645910; doi:10.1128/msphere.00432-25)
Supplement: Figure S1 — Expression of PSMα and Teg41 transcripts in different S. aureus backgrounds. [file msphere.00432-25-s0001.pdf]

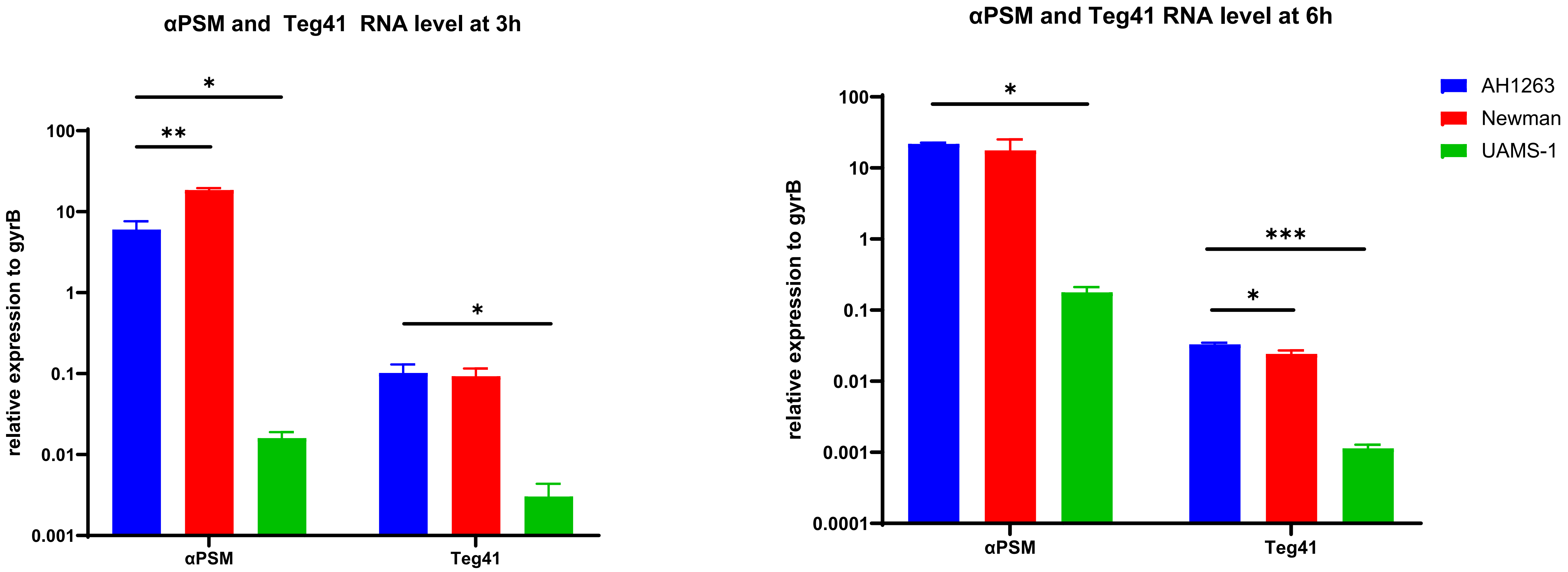

Figure S1. Expression of αPSM and Teg41 transcripts in different *S. aureus* backgrounds.
RNAs were extracted at 3 h and 6 h, and RT-qPCR was used to monitor RNA levels in AH1263 (blue), Newnan (red), and UAMS-1 (green). Expression levels were normalized to *gyrB*. Statistical significance was determined by one-way ANOVA followed by Dunnett's multiple comparison test: * $P < 0.05$, ** $P < 0.01$, and *** $P < 0.001$.
