# Supplementary material for: Analysis of Teg41 and PSMα promoter activity using a divergent fluorescent reporter plasmid
Source: mSphere. 2025 Oct 31;10(11):e00432-25. doi: 10.1128/msphere.00432-25 (PMC12645910; doi:10.1128/msphere.00432-25)
Supplement: Figure S2 — Teg41 expression in phosphomimetic mutants analyzed by RNA-seq. [file msphere.00432-25-s0002.pdf]

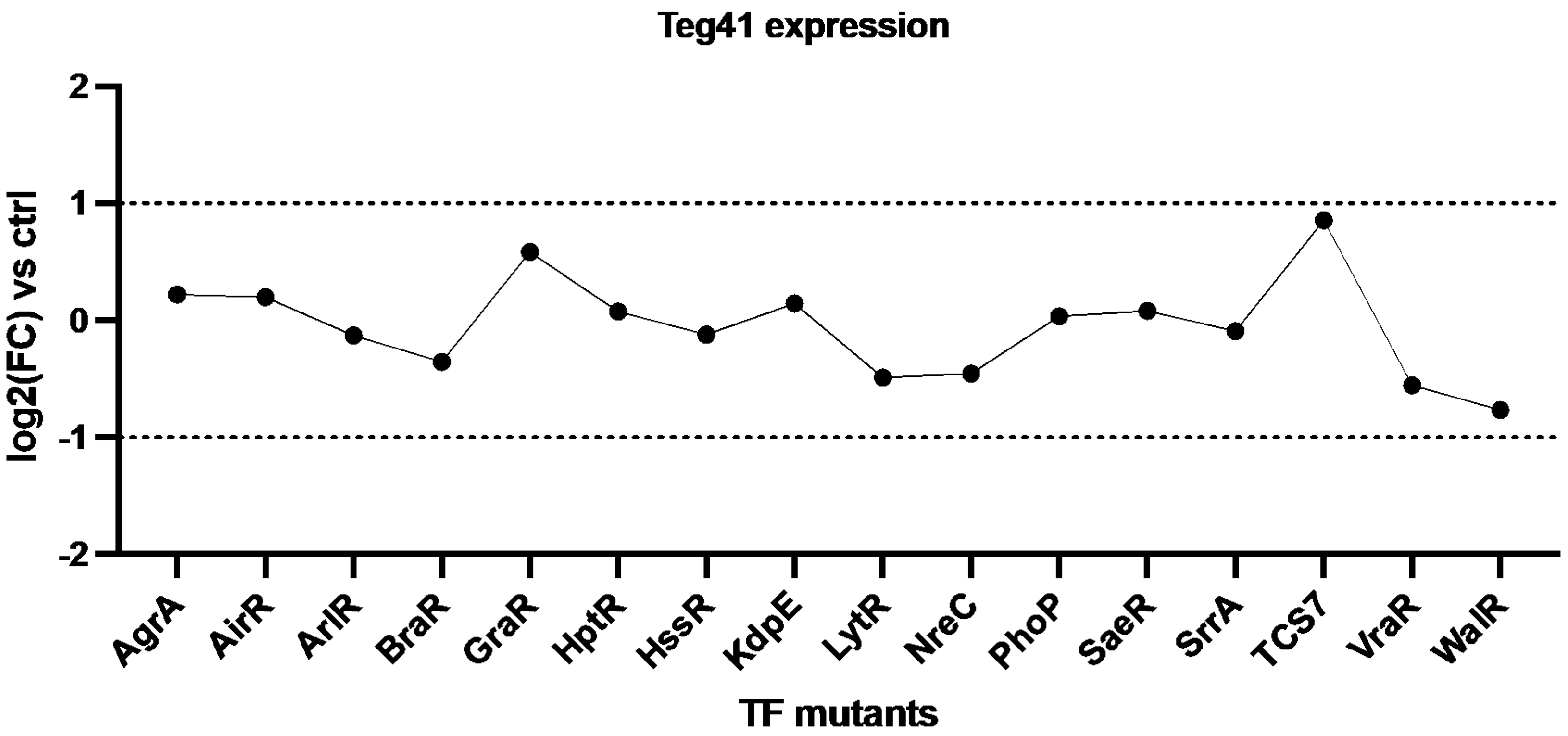

Figure S2. Teg41 expression in phosphomimetic mutants analyzed by RNA-seq.
Reads were mapped against *S. aureus* USA300 (accession CP000255.1) using Bowtie2 and read counts for each gene were obtained with FeatureCounts. Expression levels were normalized across conditions using DESeq2, and $\log_2$ fold changes were computed relative to the control (wild-type *S. aureus*). Dashed lines indicate $\log_2$ fold change > |1|.
