# Supplementary material for: Analysis of Teg41 and PSMα promoter activity using a divergent fluorescent reporter plasmid
Source: mSphere. 2025 Oct 31;10(11):e00432-25. doi: 10.1128/msphere.00432-25 (PMC12645910; doi:10.1128/msphere.00432-25)
Supplement: Figure S3 — Teg41 expression across different stimuli. [file msphere.00432-25-s0003.pdf]

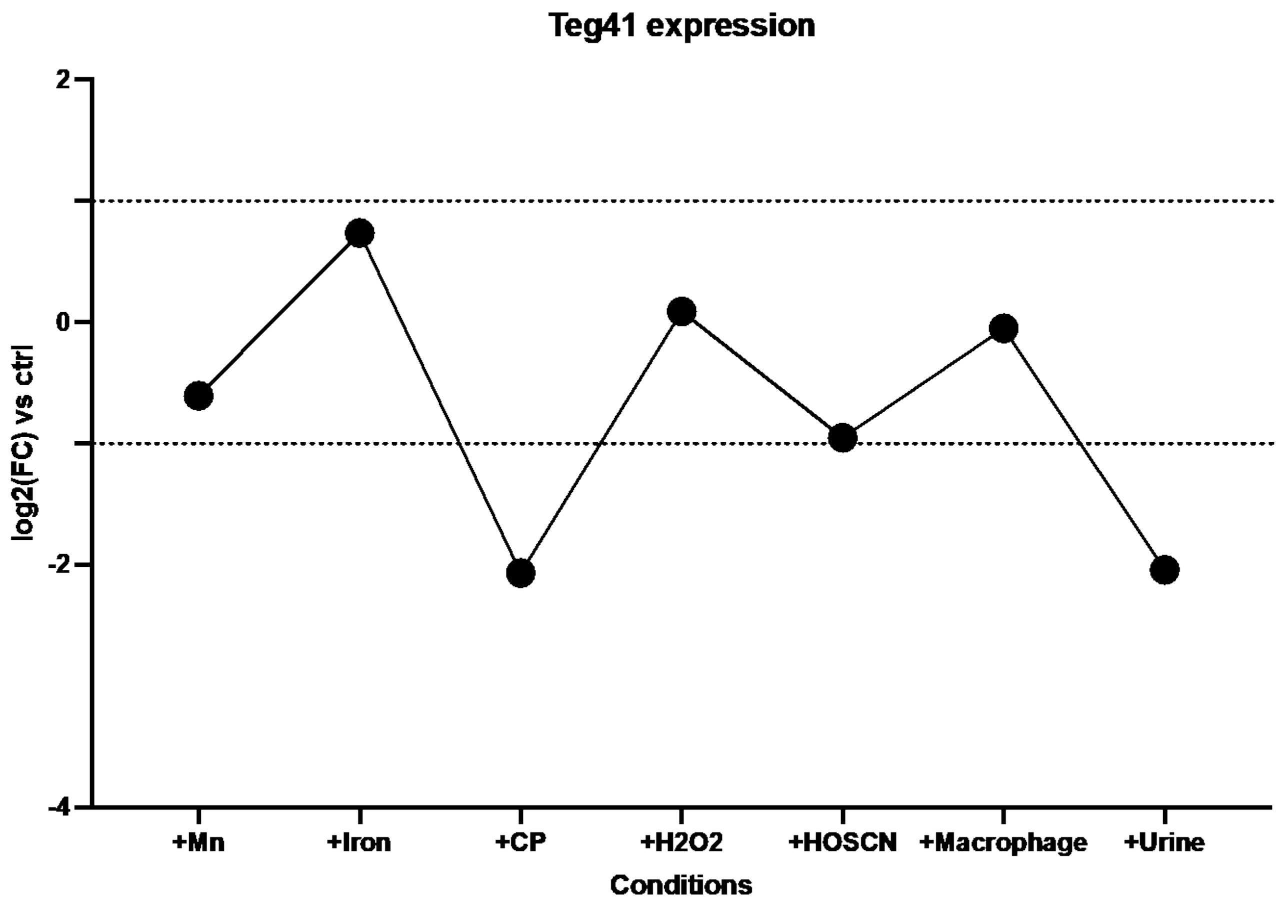

Figure S3. Teg41 expression across different stimuli.
Reads were mapped against *S. aureus* USA300 (accession CP000255.1) using Bowtie2 and read counts for each gene were obtained with FeatureCounts. Expression levels were normalized across conditions using DESeq2, and log_2 fold changes were computed relative to the control (wild-type *S. aureus*). Dashed lines indicate log_2 fold change > |1|.
