# Supplementary material for: Analysis of Teg41 and PSMα promoter activity using a divergent fluorescent reporter plasmid
Source: mSphere. 2025 Oct 31;10(11):e00432-25. doi: 10.1128/msphere.00432-25 (PMC12645910; doi:10.1128/msphere.00432-25)
Supplement: Figure S4 — Relationship between fluorescence intensity and bacterial growth for Teg41 and psmα promoters in SCFM and RPMI. [file msphere.00432-25-s0004.pdf]

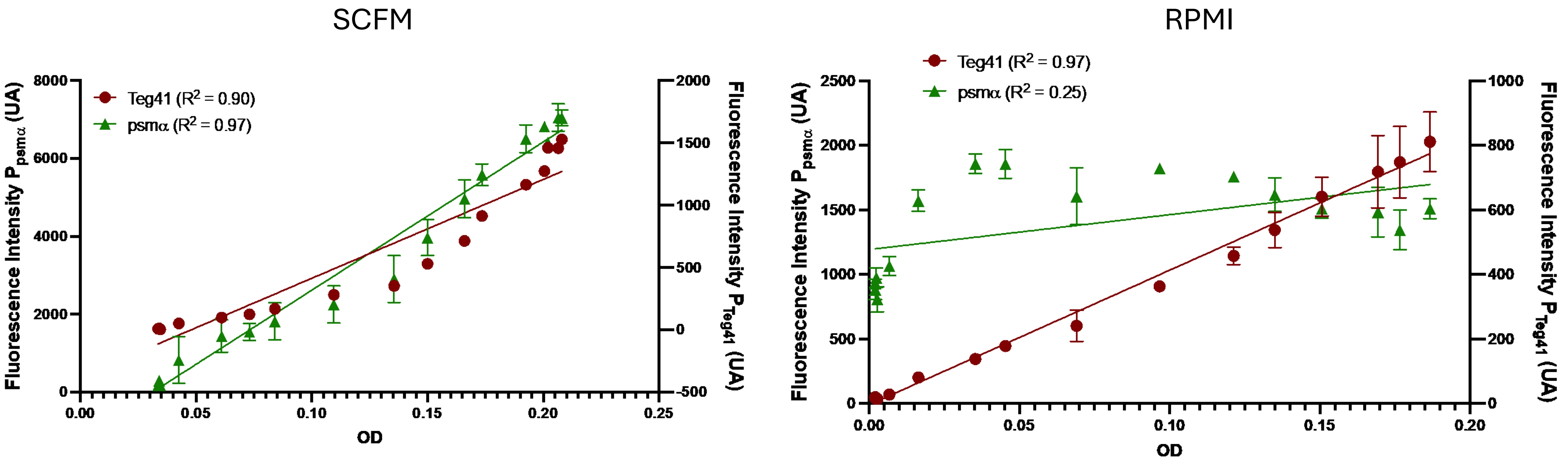

Figure S4. Relationship between fluorescence intensity and bacterial growth for Teg41 (right axis) and *psma* promoters (left axis) in SCFM (right panel) and RPMI (left panel). Strains were grown at 37 °C in 200 µl of media for 16 h in a 96-well plate with shaking. OD_{600} and fluorescence intensity (488/507 nm for GFP and 587/610 nm for mCherry) were recorded every 10 min using the BioTek Synergy H1 plate reader (Agilent). Raw fluorescence data were plotted against bacterial OD_{600}, and simple linear regression was applied (solid lines).
