# Supplementary material for: Analysis of Teg41 and PSMα promoter activity using a divergent fluorescent reporter plasmid
Source: mSphere. 2025 Oct 31;10(11):e00432-25. doi: 10.1128/msphere.00432-25 (PMC12645910; doi:10.1128/msphere.00432-25)
Supplement: Figure S5 — Coordinated expression of Teg41 and psmα in S. aureus. [file msphere.00432-25-s0005.pdf]

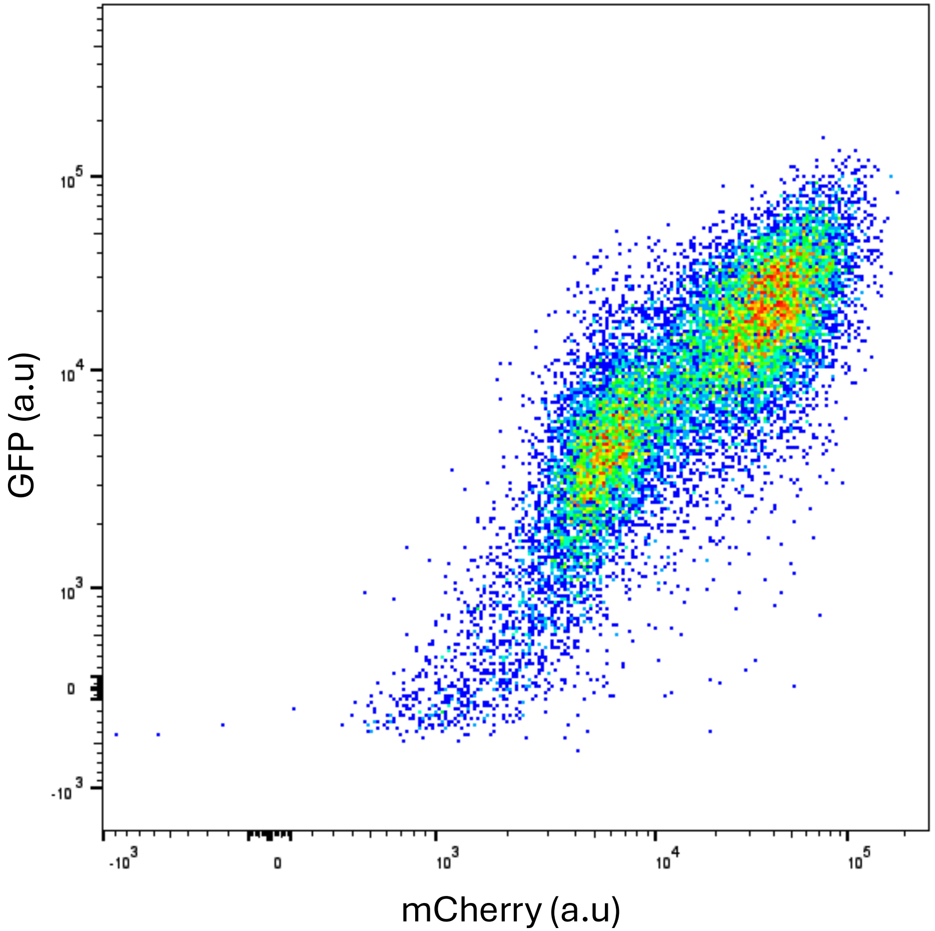


**Figure S5. Coordinated expression of Teg41 and psmα in *S. aureus*.** Flow cytometry analysis of wild-type *S. aureus* (WT) carrying plasmid pPRB4, grown in TSB for 3 h, reveals that cells with high Teg41 expression (mCherry, x-axis) also exhibit elevated psmα expression (GFP, y-axis). This suggests a positive correlation between Teg41 and psmα transcriptional activity.
